# Facilitating Stable Representations: Serial Dependence in Vision

**DOI:** 10.1371/journal.pone.0016701

**Published:** 2011-01-31

**Authors:** Jennifer E. Corbett, Jason Fischer, David Whitney

**Affiliations:** 1 Department of Cognitive, Linguistic, and Psychological Sciences, Brown University, Providence, Rhode Island, United States of America; 2 Department of Psychology, University of California, Berkeley, California, United States of America; University of Michigan, United States of America

## Abstract

We tested whether the intervening time between multiple glances influences the independence of the resulting visual percepts. Observers estimated how many dots were present in brief displays that repeated one, two, three, four, or a random number of trials later. Estimates made farther apart in time were more independent, and thus carried more information about the stimulus when combined. In addition, estimates from different visual field locations were more independent than estimates from the same location. Our results reveal a retinotopic serial dependence in visual numerosity estimates, which may be a mechanism for maintaining the continuity of visual perception in a noisy environment.

## Introduction

We experience the visual world as stable and continuous -objects retain their identities in the midst of eye movements, motion, and visual noise. To facilitate such continuity, the visual system may be inclined toward autocorrelation of perception in time, such that the percept at a given moment is biased toward what was perceived at previous moments. In this view, making the current percept dependent on past perceptual decisions could serve to maintain the continuity of objects over time, instead of continually generating new, independent percepts leading to moment-to-moment fluctuations in perceived object properties or identity. It is usually safe to assume that a currently viewed object will still be present a moment from now, rather than popping out of existence or being replaced by a different object. For example, it would be nearly impossible to drive a car on a rainy day if the rain on the windshield (visual noise) caused street signs to jitter in color, position, or even identity.

On the other hand, it would also be impossible to drive at all without somehow integrating new information, such as the changing size of approaching signs. Although perceptual autocorrelation would confer the advantage of *continuity*, it would also come at a cost when detecting new stimuli, as autocorrelation means less independence in successive visual samples, and thus reduced perceptual *accuracy* or sensitivity. In other words, when information is integrated over successive samples, maximally independent samples result in a higher ultimate signal-to-noise ratio.

It is not clear where the optimal balance lies between the trade-offs of the benefits of serial dependence (scene and object continuity) and the benefits of serial independence (sensitivity or perceptual accuracy) over successive perceptual episodes. This raises the intriguing possibility that when performing a task which benefits from integrating multiple samples, visual estimates made further apart in time will be more independent, and thus more informative (accurate) when taken together.

There is indeed evidence that human behavior exhibits substantial autocorrelation in time, even when it is not intended. For example, when we attempt to generate a “random” sequence of numbers, we cannot avoid mutual dependence in our successive responses (see [1] for a review of 15 such studies), to the extent that it is only acceptable for a computer, and not a human, to perform this task in modern experimental designs. A recent study by Vul and Pashler [2] investigated the question of how independent multiple guesses were when subjects responded to matter-of-fact questions, such as, “Saudi Arabia consumes what percentage of the oil it produces?” They found that guesses became more independent with greater intervening delay; when half of the participants in the study made a second guess to the same question a few weeks after the first, their first and second responses were more independent than the first and second responses of the other half of participants who made a second guess right away. Their results showed that while there was autocorrelation in subjects' responses over the short-term, at longer delays, subjects were able to make more independent estimates, which were more informative when taken together.

There is also evidence that autocorrelation of successive visual percepts underlies our ability to form implicit memory traces that guide subsequent perception. For example, Maljkovic and Nakayama [3] demonstrated that “priming of pop-out” occurs when observers respond to one feature of a stimulus set (shape or Vernier offset), but are facilitated by the repetition of a task-irrelevant pop-out feature in the set (color or spatial frequency). Even though the task requires no knowledge about the previous trial, or more importantly, of the pop-out feature on *any* trial, the pop-out feature exerts substantial influence on subsequent performance, whereas the task-relevant feature has no such effect. This trace of repetition priming for pop-out features diminishes over several seconds, is also manifest to a lesser degree by inhibitory effects of incongruent pop-out features on responses in successive trials, and cannot be willfully overcome. Along these lines, visual context is also implicitly learned and similarly guides subsequent perception, such that targets appearing in learned or repeated display configurations are detected more quickly than the same targets appearing in novel configurations [4].

### The Present Study

We set out to characterize serial dependence at the level of basic visual perception. Are sequential perceptual episodes correlated, and if so, do they become more independent, and therefore more useful, with greater intervening delay? Toward these ends, we presented subjects with arrays of dots and asked them to estimate how many were present in each display. Their responses became more independent with increasing delays -even delays of several seconds, indicating that successive perceptual episodes are dependent in a fashion that falls off with time. Additionally, subjects' responses were more independent across visual fields (Left/Right) than within the same visual field. This reveals that there is retinotopic specificity to the processes that mediate perceptual integration. The decreasing sequential dependence of visual percepts we observed is analogous to a form of implicit visual short-term memory, and provides insight into how perceptions at any one moment are influenced by visual experiences over the past few seconds.

## Methods

### Experiment 1: Fixed dot patterns

#### Participants

16 students at the University of California, Davis (9 women and 7 men, aged 19–38) with normal or corrected-to-normal vision voluntarily participated in a two-hour session. All participants gave written informed consent prior to the start of the experiment. The University of California, Davis's Institutional Review Board approved all procedures and protocols.

#### Stimuli and Apparatus

**Figure 1:**
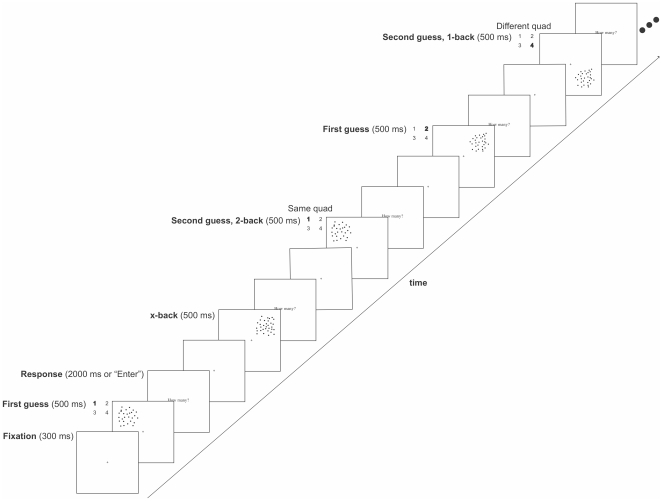
A sample sequence in Experiment 1 (in this example, a 2-back sequence is followed by a 1-back sequence). Each trial began with a fixation cross presented at the center of the screen for 300 ms. Next, one of the 21 possible patterns of 25 to 45 dots was chosen at random, and presented in one of the four quadrants for 500 ms (27 dots are presented in the top left quadrant in this example). After each pattern, the response prompt, “How many?” appeared in 33-point Times font at the center of the screen, either until the participant responded, or for 2000 ms, whichever came first. Immediately afterwards, the fixation cross re-appeared for 300 ms, and then the next pattern was presented. In this example, the next pattern was an intervening *x-back* trial with 32 dots presented in the top right quadrant, followed by the *2-back* re-presentation of the original pattern of 27 dots. Next, an initial *n-back* display of 29 dots was presented in the top right quadrant, immediately followed by a *1-back* re-presentation of the same pattern of 29 dots in the lower right quadrant.

For all participants in Experiment 1, the stimuli were the same 21 patterns of 25 to 45 black dots presented on a white background. The positions of the individual dots composing the displays were randomized on every presentation. Presentation 12.1® software (www.neurobs.com) controlled all the display, timing, and response functions. Participants were seated relative to the center of the monitor, restrained by a combination chin-and-head rest. Viewed from approximately 57 cm, individual dots subtended 0.58° of visual angle, and an entire display of dots subtended approximately 8° of visual angle. To make each of the 21 patterns, the corresponding number of individual dots were positioned within an imaginary 8×8 grid, and then randomly jittered by −0.28° to +0.28° of visual angle, both in the horizontal and vertical directions, respectively. Displays appeared in one of four possible visual quadrants (top left, top right, bottom left, or bottom right), defined relative to the horizontal and vertical meridians, centered at a diagonal eccentricity of approximately 7° of visual angle from the center of the screen, as illustrated in Figure 1.

#### Procedure

As the sample sequence in Figure 1 illustrates, trials began with a 1° of visual angle fixation cross presented at the center of the screen for 300 ms. Next, one of the 21 possible patterns of 25 to 45 dots was chosen at random, and presented in one of the four quadrants for 500 ms. After each pattern, the response prompt, “How many?” appeared in 33-point Times font at the center of the screen, either until the participant responded using the number keys on a computer keyboard and pressed the “Enter” key to advance to the next display, or for 2000 ms, whichever came first. Only responses made within this 2000 ms window were included in the experimental analysis. Immediately afterwards, fixation re-appeared for 300 ms and then the next pattern was presented.

#### n-back and x-back displays

We manipulated the delay between two presentations of the same pattern by varying the number of intervening stimulus displays, such that an initial pattern of 25 to 45 dots, chosen at random, was repeated n = 1, 2, 3, or 4 stimulus displays later (n-back). The number of dots in each of the intervening displays was randomly selected, but restricted to be different from the number of dots in the n-back pair. We call these intervening displays “x-back” trials, as they were not part of any n-back pairing. Each type of display (n-back or x-back) could appear in any one of the four quadrants of the screen (top left, top right, bottom left, or bottom right). This design yielded 64 experimental combinations, or sequences (4 n-back levels * 4 initial quadrants * 4 n-back quadrants). Each sequence was presented twice in each of five experimental blocks, for a total of 10 times each over the course of the experiment (roughly 2,240 trials per subject  = 640 initial displays +640 corresponding n-back displays + an average of 1.5 * 640 interspersed x-back displays). Displays were presented continuously, such that fixation appeared, followed by the stimulus display, followed by the response prompt, the next fixation, stimulus display, response prompt, and so on. Therefore, participants saw a stream of consecutive trials such that when questioned afterwards, they reported being unaware of specific sequences or the n-back relationship between the first and second presentations of a given pattern.

A sample 2-back sequence followed by a 1-back sequence is illustrated in Figure 1 . In this example display, an initial n-back display of 27 dots appeared in the top left quadrant, then in the intervening x-back trial, 32 dots appeared in the top right quadrant, followed by the 2-back re-presentation of the original pattern of 27 dots. Next, an initial n-back display of 29 dots was presented in the top right quadrant, immediately followed by a 1-back re-presentation of the same pattern of 29 dots in the lower right quadrant.

#### Analysis of n-backs

To measure the degree of independence between response pairs for each subject, we computed the error of the average of two estimates of the same pattern, as a function of the number of intervening displays (n-back).

#### Analysis of x-backs

Two estimates of the same x-back display (identical patterns) were randomly paired, regardless of when each was presented during the course of the experiment. Therefore, x-back pairs could contain two estimates of the same pattern presented at any times during the course of the experiment, from two temporally contiguous presentations, as in 1-back trials, or one presentation from the beginning of the experiment and one from the end of the experiment. Because x-backs were randomly paired, they served as an approximate index of the upper bound of variability, or independence possible between two of a given participant's estimates. For this reason, we included these trials as a 5th level of n-back in the corresponding ANOVA.

### Experiment 2: Random dot positions

#### Participants

16 trained observers (5 women and 11 men, aged 19–39) with normal or corrected-to-normal vision voluntarily participated in a two-hour session. All participants gave written informed consent prior to the start of the experiment. The University of California, Davis's Institutional Review Board approved all procedures and protocols.

#### Stimuli

The stimuli used in Experiment 2 were identical to those used in Experiment 1, except that now the positions of the individual dots composing the displays of 25 to 45 dots were randomized on every presentation. On each trial, the appropriate number of dots were positioned randomly within an imaginary 8×8 grid, then randomly jittered by −0.28° to +0.28° of visual angle in both horizontal and vertical directions, meaning that there were now approximately 2,240 unique patterns presented over the course of the experiment (640 initial displays +640 corresponding n-back displays + an average of 1.5 * 640 interspersed x-back displays), to help eliminate possible effects of spatial configuration from the 21 repeating patterns that may have affected the results of Experiment 1.

#### Task, Apparatus, and Procedure

All other methods in Experiment 2 were identical to those in Experiment 1 with the above exception that the positions of individual dots were randomized on each trial to eliminate repeated patterns.

## Results

### Experiment 1

In the first experiment, we presented subjects with brief displays of 25 to 45 dots, and asked them to estimate how many dots were in each display. The spatial arrangement of the dots in each display was fixed, resulting in 21 different dot patterns. Similar to the approach of Vul and Pashler [2], we measured the degree of independence between a pair of estimates by computing how much of an accuracy improvement was afforded by averaging the responses, versus considering them separately.

By this definition of independence, more independent estimates carry more mutual information when taken together. Therefore, our measure of independence speaks directly to the central question of the study: Is it more informative to make multiple visual estimates further apart in time? Although the correlation between the first and second estimates (autoregression) could be used as an alternative definition of serial dependence, this is not exactly the same as the measure of independence we calculate here. We sought to measure the amount of information gained by combining successive estimates (i.e., How useful is it to take another glance at the stimulus?). Our approach of measuring the improvement afforded by averaging multiple estimates with variable intervening delay is thus a more direct measure of the outcome of interest. If multiple perceptual episodes do become more independent over time, then averaging pairs of estimates should yield more improvement as the number of intervening trials increases.

#### Average versus individual first and second errors

To compare the amount of information gained from a second estimate of the same pattern to the information obtained from either corresponding single estimate, we calculated:

1. The average Mean Squared Error (MSE) of the first and second estimates (Est) relative to the actual number of dots displayed (nDots) for each response pair: *Average MSE of individual estimates  =  ((Est1 - nDots)^2^ + (Est2 - nDots)^2^))/2,*

2. The MSE of the average of the two estimates: MSE of the average of estimates  =  (Est1 + Est2)/2)- nDots)^2^,

3. Then, the amount of improvement in MSE from averaging the two estimates versus the MSE of either estimate: Improvement from averaging  =  Average MSE of individual estimates - MSE of the average of estimates.

#### N-back

**Figure 2:**
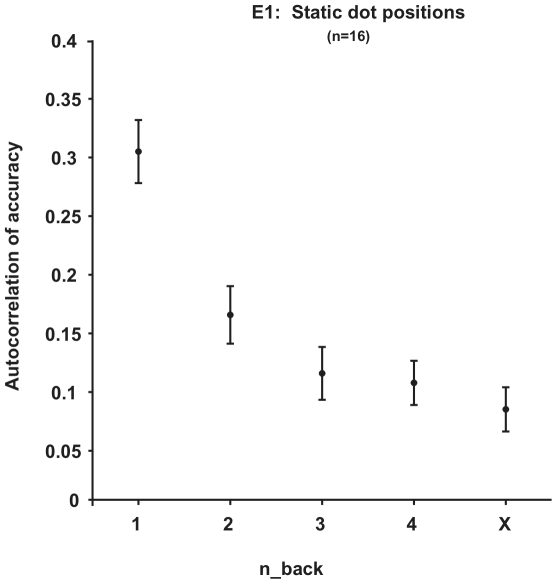
Experiment 1, Fixed dot positions, Autocorrelation. Subjects' responses were serially correlated as a function of n-back (error bars  = ±1 SEM).

Subjects' responses were serially correlated. Figure 2 shows the correlation between subjects' first and second responses (autocorrelation) as a function of n-back. The autocorrelation plot is a reasonable way to estimate the serial dependence in responses, but it does not capture the accuracy of subjects' responses; autocorrelation and accuracy can be orthogonal. Therefore, to capture how independent and collectively informative subjects' pairs of responses were, we calculated the improvement in accuracy afforded by averaging the responses together.

**Figure 3:**
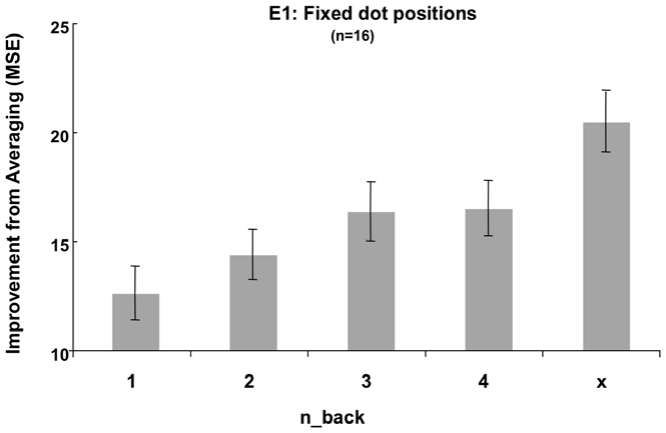
Experiment 1, Fixed dot positions, n-back. The improvement from averaging two estimates (MSE) increased with greater intervening delays (n-back), indicating that responses became more independent over time (error bars  =  ±1 SEM).

As outlined in the Introduction, the more accurate the average of two estimates compared to either single estimate, the more independent they are. Any such increase in independence between response pairs corresponds to an increase in the amount of information to be gained from obtaining a second estimate. Figure 3 shows this improvement in MSE gained from averaging paired first and second estimates relative to the individual MSEs of the estimates, for each level of n-back. The improvement from averaging two estimates increased with greater intervening delays, indicating that responses became more independent over time. This pattern was confirmed by a 5(n-back) * 2(Location) repeated-measures ANOVA on subjects' overall improvement from averaging (MSE). This analysis revealed a significant main effect of n-back (*F*(4,60)  = 12.791, *MSE*  = 275.046, *p*<.001, *η^2^* = .46), as well as a significant linear increase in the improvement from averaging with increasing n-back, from 1-back to x-back (*F*(1,15)  = 31.493, *MSE*  = 997.349, *p*<.001, *η^2^* = .68).

#### Location

**Figure 4:**
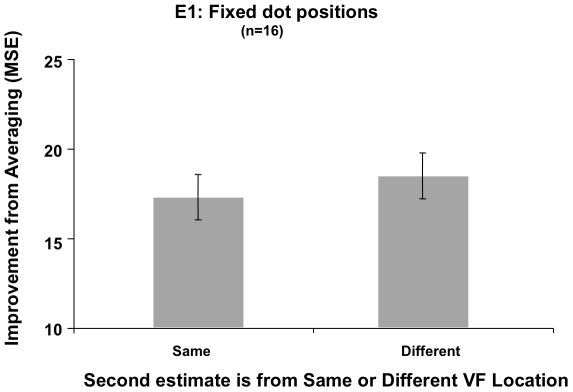
Experiment 1, Fixed dot positions, Location. The improvement from averaging two estimates from different versus the same locations was marginally significant, suggesting that pairs of estimates taken from different visual field locations were somewhat more independent than estimates paired from the same location (error bars  =  ±1 SEM).

Estimates from different visual field locations were somewhat more independent than estimates paired from the same location, as the marginally significant small effect of visual field location accounted for about 22% of the variance in the repeated-measures ANOVA on participants' improvement from averaging estimates over the accuracy of either single corresponding estimate (*F*(1,15 = 4.142, *MSE* = 82.842, *p* = .06, *η^2^* = .22; Figure 4). Finally, the ANOVA showed no significant interaction between n-back and visual field location (*p* = .203, *η^2^* = .09), suggesting that no single n-back level drove the marginal effect of visual field location observed in Experiment 1. Note that we collapsed our analysis of location over the upper and lower quadrants of each visual field in the omnibus ANOVA, as although we were concerned with the spatial dependence of successive visual percepts, we wanted to capitalize on the known asymmetry in pattern recognition between the left and right visual fields (e.g. [5]), and we did not wish to contaminate our results with any other differences in performance between upper and lower visual fields (e.g. [6]).

### Experiment 2

We conducted a second experiment to investigate whether the fixed spatial arrangement of the dots (repeated patterns) influenced the serial dependence of observers' reports in Experiment 1. Experiment 2 was identical to Experiment 1 in all respects, except that the positions of all dots were randomized in every stimulus display. In other words, whereas both the number and positions of the dots in each stimulus display were fixed in Experiment 1, only the number of dots remained constant for the 21 possible experimental stimulus combinations in Experiment 2. If presenting the same 21 patterns over the course Experiment 1 caused the observed n-back and visual field effects, then randomizing the locations of the individual dots presented on each display should eliminate any such effects of repeated spatial configurations and familiarity in Experiment 2.

#### n-back

**Figure 5:**
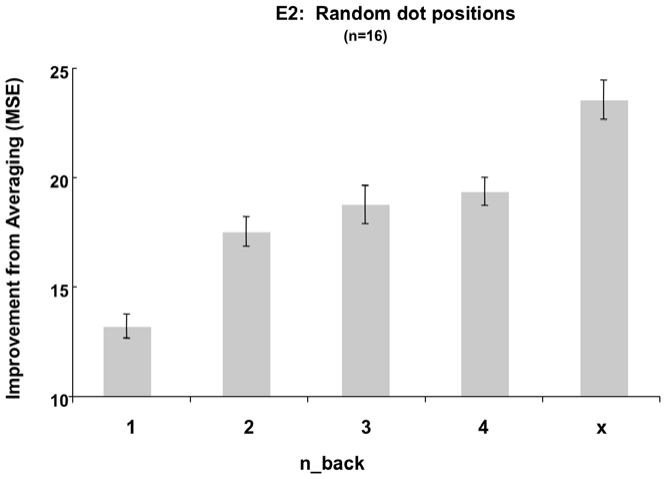
Experiment 2, Random dot positions, n-back. As in Experiment 1, the improvement from averaging two estimates (MSE) increased with greater intervening delays (n-back), indicating that responses became more independent over time (error bars  =  ±1 SEM).

Subjects' first and second responses were again serially correlated as a function of n-back. Bootstrapping the difference between the autoregression plots of Experiments 1 and 2 at each n-back 1000 times for the six observers who participated in both Experiments 1 and 2 revealed no significant difference in the autocorrelations over the two experiments (all *p*'s>1). More importantly, to quantify the amount of information to be gained from obtaining a second estimate in Experiment 2, we conducted a 5(n-back) * 2(Location) repeated-measures ANOVA on subjects' overall improvement from averaging estimates over the accuracy of either single corresponding estimate, just as in Experiment 1. This analysis again revealed a significant effect of n-back (*F*(4,60)  = 23.286, *MSE* = 507.254, *p*<.001, *η^2^* = .61), and a significant linear trend on the amount of improvement from averaging (MSE) with increasing n-back, from 1-back to x-back (*F*(1,15)  = 39.474, *MSE* = 1853.669, *p*<.001, *η^2^* = .73; Figure 5).

#### Location

**Figure 6:**
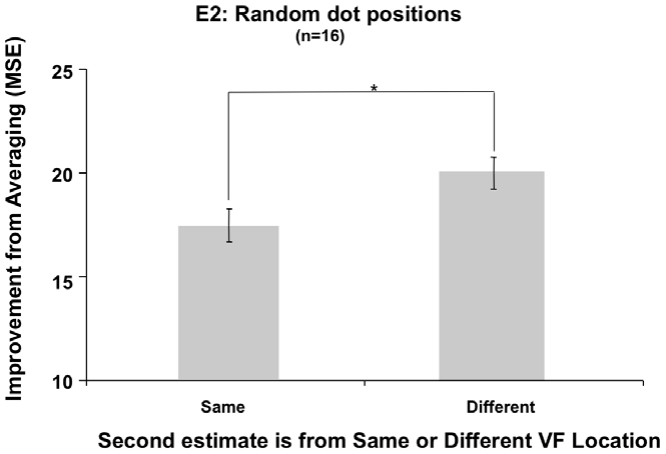
Experiment 2, Random dot positions, Location. The improvement from averaging two estimates from different versus the same locations was now significant, supporting a retinotopically specific dependence between pairs of successive estimates (error bars  =  ±1 SEM).

As illustrated in Figure 6, in Experiment 2, when the position of dots composing individual displays were randomized on each trial to eliminate the effects of repeated spatial configuration or patterns, there was now a significant effect of visual field location that accounted for over half of the variance in the amount of improvement from averaging two of a given participant's estimates over the accuracy of either of the individual estimates (*F*(1,15)  = 18.928, *MSE* = 329.898, *p* = .001, *η^2^* = .56). There was again no significant interaction between n-back and visual field location (*p* = .554, *η^2^* = .05), providing no evidence that any single n-back level was responsible for the observed n-back and visual field effects again observed in Experiment 2.

## Discussion

### Experiment 1

The results demonstrated that judgments of patterns are serially dependent; magnitude estimations for a particular pattern depended on whether the pattern was previously seen within the last 4+ trials. This serial dependence extended over relatively long delays and intervening trials, suggesting that pattern perception is not independent over multiple viewings.

One concern is that the spatial arrangement of the dot patterns was fixed, and subjects may have learned the dot patterns or used information about the spatial arrangement per se, rather than estimating the number of dots. Further, the experiment revealed a marginally significant effect of the visual field manipulation that accounted for a small, but substantial portion of the variance in the extent to which accuracy improved from averaging two of an observer's subsequent estimates. This effect may have been suppressed in Experiment 1 because the dots always appeared in a fixed position specific for each of the individual 21 stimulus displays. If the dot positions had been randomized for every stimulus display, so as not to introduce pattern repetition or similar contextual cues, we might expect a stronger visual field effect. To address these concerns, a second experiment randomized the dot positions to reduce pattern-based cues and increase uncertainty about the stimuli.

### Experiment 2

Experiment 2 yielded a very similar pattern of n-back results to Experiment 1; there was a serial dependence in observers' magnitude estimates, and increasing the number of intervening patterns between first and second estimates reduced this serial dependence. As illustrated in Figure 5, the amount of improvement obtained from averaging two estimates from a given participant versus the accuracy of either corresponding estimate increased from the 1-back to the x-back level, as evidenced by the main effect and corresponding linear trend of n-back.

In contrast to Experiment 1, when the dot positions were randomized in Experiment 2 to allow for a unique pattern to be presented on every display, there was a significant effect of visual field, such that estimates made within a visual field were more serially dependent than estimates from different visual fields. This effect was more than twice the size of the same trend that was apparent, but only marginally significant in Experiment 1, when the dots were presented at fixed locations specific to each of the 21 possible stimuli. The difference in the results of the two experiments may reveal a difference in the way subjects performed the task; the fixed dot configurations could, in principle, be judged by learning visual patterns or context (e.g. [4]), whereas when the positions were scrambled, subjects had to judge the numerosity (or perhaps density; see General Discussion). The additional cues provided by fixed dot patterns in Experiment 1 could have led to differences in the spatial aspect of serial dependence as well.

### General Discussion

The experiments here demonstrate that sequential visual percepts are serially dependent. In particular, the perceived number of an array of objects depends on the perceived number of objects in previous displays. This serial dependence of visual percepts works over relatively long delays, and intervening images. The results demonstrate a form of implicit visual short-term memory, and provide a method of measuring this without requiring an explicit memory judgment or recall.

A possible alternative explanation for our results is that the dependence in subjects' successive estimates is a result of autocorrelation of *responses*, rather than of perception. That is, subjects may have a tendency to give the same or similar responses on subsequent trials, regardless of the stimulus, or even modality. However, such a tendency cannot account for our results here, as subjects' estimates were more independent when the stimuli appeared in different regions of the visual field, but autocorrelation in responses alone would produce dependence across all regions of the visual field in a stimulus-independent fashion. Instead, our results point to serial dependence in the underlying perceptual mechanisms responsible for subjects' numerosity judgments.

We can also rule out the alternative that our results are due to perceptual learning. Although it is possible that subjects may have learned the stimuli, especially in Experiment 1 where the patterns were fixed, an overall learning effect would not predict a serial dependence that is delay (n-back) dependent. Further, the error distributions around estimates did not change significantly over the course of the experiments, and there was no feedback in the experiments, suggesting that statistical learning effects (e.g. [7], [8]) were not responsible for the results.

#### Relation to the visual sense of number

Subjects in our experiments judged the number of dots in the displays. This could rely on mechanisms that represent numerosity per se, texture density, or a combination. In any case, our results show that pattern recognition (of number or density) is position sensitive, and has an implicit memory trace. Our results could support Burr and Ross's recent argument for a visual number sense [9]-[11]. Specifically, we found that the serial dependence of numerosity judgments was location-specific (stronger within, than across visual fields). This could be consistent with a retinotopic (or spatiotopic) source of numerosity judgments [9], [10]. On the other hand, numerosity judgments could depend, at least in part, on a texture density cue [12].

#### Relation to priming and adaptation

The serial dependence uncovered in the present investigation may be related to findings reviewed by Pearson and Brascamp [13], in which bistable stimuli can be sequentially primed, such that a single percept becomes dominant (i.e., hysteresis-like effects). It may also be related to Burr and Ross's reports [9], [10] that adaptation to numerosity negatively biases subsequent estimates (i.e., a negative aftereffect). To examine whether the same type of implicit sensory memory suggested by these results may also underlie our findings, future investigations must be conducted to resolve several key differences between these studies and the present investigation of numerosity perception over time. First, our stimulus is not ambiguous. Previous results, outlined by Pearson and Brascamp [13], show a near threshold effect on the perceptual dominance of an ambiguous figure, usually in rivalry displays, in-line with hysteresis effects consistently reported over the years. Although there is some uncertainty in our number task, our stimulus is not bistable, only one pattern is visible on each trial, and the stimulus visibility is not degraded with noise or other competing patterns. Second, our task is not dichotomous, unlike hysteresis and negative aftereffect studies. Further, there was no sequential repulsion of numerosity estimates, suggesting that a negative aftereffect did not produce the results here. Finally, we are not reporting a “dominant” percept that persists or oscillates with one other stimulus interpretation. Instead, we show that the error subjects make tends to be affected by previous trials with the same stimulus type, even when there are multiple intervening stimuli.

#### Scope of implicit memory and serial dependence of visual perception

The serial dependence in visual magnitude estimates occurred over relatively long delays and several intervening images. This suggests that the implicit memory trace of a visual pattern could influence the perception of subsequent retinal images, perhaps even over several eye movements and in natural viewing conditions. The disadvantage of serially dependent recognition is the possibility that it might work against temporal integration mechanisms (e.g., of motion, orientation, color, faces, etc); temporal integration would be most effective with more independently coded patterns. For example, serial dependence may be a factor in change blindness, or our poor ability to detect even large and salient changes in real-world events (e.g. [14], [15]). On the other hand, there are potential benefits of these kinds of implicit memory traces. For example, if spatiotopically specific, the serial dependence could help maintain object representations across eye movements. Therefore, it might help with recognition and memory of patterns, objects, and scenes, and add to perceptual stability in the face of constant eye movements.

To address the possible costs and benefits, it is important that future studies measure the time course of this serial dependence in vision, including the effect of stimulus duration. Stimulus duration is a particularly important parameter because it mediates priming (facilitation) and adaptation (suppression) in the similar, hysteresis-like effects previously mentioned (see [16] for a discussion). In any case, the serial dependence in visual judgments over time may reflect an adaptive mechanism to arbitrate perceptual demands for independence (accuracy) versus continuity and stability.

#### A novel measure of implicit visual short-term memory

Our paradigm provides a new, implicit measure of visual short term-memory. Further, the degree to which a delay between guesses helps improve the average estimate allows for a measure of memory decay, or a “forgetting curve,” in that guesses become more independent, or variant, as they are separated further in time. Unlike reports given in typical short-term memory experiments using change detection [14], [15], or multiple object tracking tasks [17], [18], this implicit measure of averaging multiple estimates allows for a measure of the contents of working memory, including information that may not be available for conscious report. In addition, it avoids the effects of requiring explicit encoding, which does not happen under normal viewing conditions (we do not actively try to remember as many objects as possible from one eye movement to another). Unlike other methods of implicit visual short-term memory, such as contextual cueing and priming of pop-out [3], [4], the effect here does not depend on a manipulated context.

### Conclusions

Overall, our findings suggest that the amount of information to be gained from a second estimate of the same stimulus increases as the time between estimates increases. In addition, second estimates taken from different areas of the visual field are more informative (independent) than estimates taken from the same location. The retinal specificity of perceptual serial dependence suggests that it may function to maintain object continuity in the midst of a noisy environment. The decay of this dependence over several trials (Figures 2 , 3 , 5) may represent an optimal balance between maintaining object continuity and maintaining an updated representation of the visual environment.
